# Robust Cell-Free Expression of Sub-Pathological and Pathological Huntingtin Exon-1 for NMR Studies. General Approaches for the Isotopic Labeling of Low-Complexity Proteins

**DOI:** 10.3390/biom10101458

**Published:** 2020-10-19

**Authors:** Anna Morató, Carlos A. Elena-Real, Matija Popovic, Aurélie Fournet, Karen Zhang, Frédéric Allemand, Nathalie Sibille, Annika Urbanek, Pau Bernadó

**Affiliations:** Centre de Biochimie Structurale (CBS), INSERM, CNRS and Université de Montpellier. 29 rue de Navacelles, 34090 Montpellier, France; anna.morato@cbs.cnrs.fr (A.M.); carlos.elena-real@cbs.cnrs.fr (C.A.E.-R.); matija.popovic.ri@gmail.com (M.P.); aurelie.fournet@cbs.cnrs.fr (A.F.); kz7@princeton.edu (K.Z.); frederic.allemand@cbs.cnrs.fr (F.A.); nathalie.sibille@cbs.cnrs.fr (N.S.)

**Keywords:** huntingtin protein, low-complexity regions, nuclear magnetic resonance (NMR), cell-free protein expression, isotopic labeling, site-specific isotopic labeling, nonsense suppression

## Abstract

The high-resolution structural study of huntingtin exon-1 (HttEx1) has long been hampered by its intrinsic properties. In addition to being prone to aggregate, HttEx1 contains low-complexity regions (LCRs) and is intrinsically disordered, ruling out several standard structural biology approaches. Here, we use a cell-free (CF) protein expression system to robustly and rapidly synthesize (sub-) pathological HttEx1. The open nature of the CF reaction allows the application of different isotopic labeling schemes, making HttEx1 amenable for nuclear magnetic resonance studies. While uniform and selective labeling facilitate the sequential assignment of HttEx1, combining CF expression with nonsense suppression allows the site-specific incorporation of a single labeled residue, making possible the detailed investigation of the LCRs. To optimize CF suppression yields, we analyze the expression and suppression kinetics, revealing that high concentrations of loaded suppressor tRNA have a negative impact on the final reaction yield. The optimized CF protein expression and suppression system is very versatile and well suited to produce challenging proteins with LCRs in order to enable the characterization of their structure and dynamics.

## 1. Introduction

Huntington’s disease (HD) is one of nine neurodegenerative disorders caused by CAG triplet repeats resulting in polyglutamine (poly-Q) expansions within proteins [1,2]. In case of HD, the causative agent is a mutant of huntingtin protein, whose poly-Q tract is located in exon-1 (HttEx1) of the ~348 kDa protein [3]. With poly-Q tract lengths surpassing the pathological threshold of 35 glutamines, huntingtin is becoming progressively more aggregation prone and forms large cytoplasmic and nuclear fibers within neurons of the striatum, triggering neurodegeneration [4,5]. Moreover, the length of the expanded poly-Q tract correlates with the age of onset and disease severity [1].

**Figure 1:**
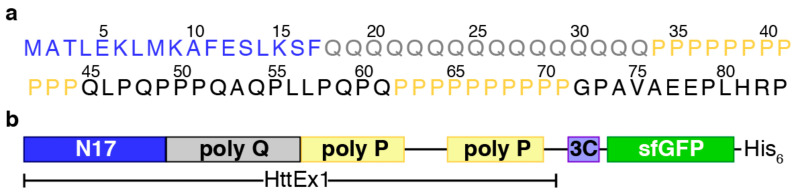
The huntingtin exon-1 construct used in this study. (**a**) Amino acid sequence of huntingtin exon-1 (HttEx1). Note that the numbering corresponds to an HttEx1 construct with 16 glutamines (H16). N17 and the homorepeats of glutamine and proline are colored in blue, gray and yellow, respectively. (**b**) Cartoon of the construct used in this study with HttEx1 fused to a 3C cleavage site, followed by superfolder green fluorescent protein (sfGFP) and the His-tag.

Strikingly, the mutant HttEx1 fragment alone suffices to reproduce HD symptoms in mice [6] and has been the construct of choice for in vivo and in vitro studies [7,8,9,10,11,12,13] (see below). The primary sequence and domain organization of sub-pathological HttEx1 are shown in Figure 1. The N-terminal domain, preceding the poly-Q tract, is composed of 17 residues (N17) and has been shown to enhance the aggregation of poly-Q tracts in vitro and in vivo [14,15]. Downstream of the poly-Q tract, HttEx1 contains a proline-rich region (PRR) with two poly-proline (poly-P) tracts of 11 and 10 consecutive prolines, respectively. While the poly-P tract has a protective effect against HttEx1 aggregation in vitro and in vivo, it is necessary for the formation of visible aggregates in cells [13,16,17,18]. Interestingly, this protective effect is directional, as N-terminal poly-P tracts do not attenuate the aggregation of poly-Q peptides [16]. N17 and the PRR also impact the aggregation pathways of HttEx1, the structure and stability of the formed fibrils, and the toxicity [13,19]. Already at the scale of the monomer, the flanking regions govern the structure and dynamics of HttEx1 [10,15,20,21].

While HD and huntingtin have been the focus of numerous investigations for decades, many aspects of the molecular mechanisms underlying the disease remain unclear. This is mostly due to the above-mentioned propensity of mutant huntingtin to form a heterogeneous mix of oligomers and aggregates [8,22]. In order to prepare sufficient amounts of soluble protein while attenuating its aggregation, Httex1 is usually expressed in fusion with solubilizing protein tags such as maltose-binding protein (MBP), glutathione S-transferase (GST), or thioredoxin (Trx) [7,8,9,23]. Depending on the type of assay, e.g., aggregation, structure determination, or interaction studies, these solubilizing tags may be removed by specific proteases. In addition to being aggregation prone, HttEx1 is highly flexible and crystallization and cryo-electron microscopy studies could not provide structural information on the poly-Q tract [21,24], leaving nuclear magnetic resonance (NMR) the only technique to obtain high-resolution data on HttEx1. However, with the repetitive nature of the Httex1 sequence, which produces highly congested spectra, traditional NMR experiments reach their limit, requiring novel strategies to support the sequential assignment and the acquisition of structural restraints [10,11,15,20,25,26,27].

Cell-free (CF) coupled transcription-translation systems based on *Escherichia coli* extracts have become an attractive alternative to conventional in vivo expression systems [28,29,30]. CF systems de-couple the cultivation of bacteria from protein expression, which is beneficial for toxic proteins and can reduce the time of the actual protein synthesis. Templates can be provided in form of DNA or RNA, and the expression of different constructs (e.g., fusions with different protein tags) can be performed in a high throughput manner [31,32]. One key feature of CF reactions is that they are open systems and can be tailored to the protein of interest. They tolerate a wide range of compounds that can be added at any time over the course of the reaction, including detergents, lipid mixtures, enzymes and other chemicals [33,34,35]. In order to produce NMR samples, CF enables many different protein-labeling strategies (uniform labeling, selective labeling and site-specific labeling) given the control over the isotopic composition of the amino acids introduced in the reaction mixture and the reduced extent of the scrambling processes [36,37,38]. Furthermore, CF production is notably less expensive regarding the use of isotopes than in vivo cultures since small reaction volumes suffice [28,29,39,40,41].

Here, we describe the development of a robust expression protocol of sub-pathological and pathological HttEx1 constructs in a CF system. Our protocol has been used to produce Httex1 with different labeling schemes, enabling the detailed structural characterization of different constructs of the protein. The optimization of coupling CF expression with the tRNA suppression strategy [42,43], which allows the site-specific isotopic labeling (SSIL) of proteins, is also described. The ensemble of our developments provides new avenues for the structural characterization of proteins hosting low-complexity regions (LCRs) [44] and, therefore, to decipher the bases of biological processes such as neurodegeneration [45] or liquid-liquid phase separation [46,47].

## 2. Materials and Methods

Unless specified otherwise, all chemicals were obtained from Sigma-Aldrich (St. Quentin Fallavier, France).

### 2.1. DNA Constructs

HttEx1 plasmids were prepared as previously described [25]. Briefly, synthetic genes coding for human HttEx1 with 16 consecutive glutamines (H16) or H16 amber suppression mutants (TAG) were ordered from Integrated DNA Technologies (IDT, Leuven, Belgium). A synthetic gene coding for HttEx1 with 46 consecutive glutamines (H46) was ordered from GeneArt^®^ (ThermoFisher Scientific, Waltham, MA, USA). Using an In-Fusion^®^ (Clontech, Mountain View, CA, USA) reaction, the synthetic genes were cloned into pIVEX 2.3d, giving rise to pIVEX HttEx1(16/46)-3C-sfGFP-His_6_ and mutants. A synthetic gene coding for *E. coli* EF-Tu with an N-terminal Strep-Tag II followed by a TEV cleavage site was obtained from IDT (Leuven, Belgium). It was subcloned into a pET11a vector using the NdeI and HindIII restriction sites by an In-Fusion^®^ reaction, giving rise to pET11a Strep-TEV-EF-Tu. All plasmids were sequenced by GENEWIZ^®^ (Leipzig, Germany).

### 2.2. Expression, Purification and Activation of EF-Tu

BL21 (DE3) cells carrying the pET11a Strep-TEV-EF-Tu plasmid were grown in ZYM medium [48] with 100 µg/µL Amp at 37 °C and 220 rpm for ~6 h. Cells were harvested by centrifugation (6000× *g*, 30 min, 4 °C). Cells were resuspended in buffer EF-Tu A (50 mM Tris pH 7.5, 300 mM NaCl, 10 mM MgCl_2_, 1 mM DTT, 0.1 mM GDP) with added protease inhibitors (c0mplete EDTA-free). Cell lysis was achieved by sonication. The lysate was clarified by centrifugation (40,000× *g*, 30 min, 4 °C) and then loaded onto a Strep-Tactin^®^ column (bed volume 5 mL, IBA Lifesciences, Göttingen, Germany) equilibrated in buffer EF-Tu A and washed with 10 CV buffer EF-Tu B (same as buffer EF-Tu A but with 1000 mM NaCl) followed by 5 CV buffer EF-Tu A. EF-Tu was eluted in 2.5 mL fractions using buffer EF-Tu A with added buffer E (IBA Lifesciences, Göttingen, Germany). EF-Tu containing fractions were detected by SDS-PAGE analysis, pooled and dialyzed against buffer EF-Tu A overnight at 4 °C. Subsequently Strep-EF-Tu was concentrated to a final concentration of 150 µM using a Vivaspin 6 concentrator (MWCO 10 kDa, Sartorius, Göttingen, Germany), aliquoted, flash-frozen and stored at −20 °C. For the described studies, the Strep-tag II was not removed.

EF-Tu activation was performed as described by Nazarenko et al. and Pleiss et al., but with slight modifications [49,50]. Briefly, activation was achieved by incubating 100 µM EF-Tu with 1x activation buffer (50 mM Tris pH 7.5, 150 mM NH_4_Cl, 20 mM MgCl_2_, 5 mM DTT, 3 mM phosphoenolpyruvate, 30−40 µg/mL pyruvate kinase (454 units/mg), 1 mM GTP) at 37 °C for 3 h. Activated EF-Tu was added to CF expression or aa-tRNA_CUA_ stability assays without further purification.

### 2.3. Lysate Preparation and CF Expression Conditions

#### 2.3.1. Lysate Preparation

All lysate batches were based on the *Escherichia coli* strain BL21 Star (DE3): RF1-CBD3, a gift from Gottfried Otting (Australian National University, Canberra, Australia) [40] and prepared as described previously [20,25].

#### 2.3.2. Standard Batch Mode CF Expression Conditions

CF protein expression was performed in batch mode [20,25,51]. The standard batch mode reaction mixture consisted of the following components: 55 mM HEPES-KOH (pH 7.5), 1.2 mM ATP, 0.8 mM each of CTP, GTP and UTP, 1.7 mM DTT, 0.175 mg/mL *E. coli* total tRNA mixture (from strain MRE600), 0.64 mM cAMP, 27.5 mM ammonium acetate, 68 µM 1-5-formyl-5,6,7,8-tetrahydrofolic acid (folinic acid), 1 mM of each of the 20 amino acids, 80 mM creatine phosphate (CP), 250 µg/mL creatine kinase (CK), plasmid (16 µg/mL) and 22.5% (*v*/*v*) S30 extract. The concentrations of magnesium acetate (5–20 mM) and potassium glutamate (60–200 mM) were adjusted for each new batch of S30 extract and HttEx1 construct.

#### 2.3.3. Optimization of CF Suppression Conditions

Two artificial suppressor tRNAs (one for glutamine and one for proline) were transcribed, purified, refolded and aminoacylated as previously described [20,25,52]. To optimize the CF reaction for nonsense suppression, different concentrations of loaded tRNA_CUA_ (0–30 µM final concentration of total tRNA_CUA_) were added to the reaction mix. Protein expression of H16Q20 or H16P34 was followed by sfGFP fluorescence using a plate reader/incubator (Gen5 v3.03.14, BioTek Instruments, Colmar, France) at 485 nm (excitation) and 528 nm (emission). Assays were carried out in a reaction volume of 50 µL dispensed in 96-well plates and incubated at 23 °C for 3.5 h. The same setup was used to investigate the effect of 0–40 µM aa-tRNA_CUA_ on a positive control, meaning the CF synthesis of H16-sfGFP without any amber codon.

#### 2.3.4. Uniform and Selective Labeling in CF Expression Systems

Uniformly or selectively labeled HttEx1 samples for NMR studies were produced at 5−10 mL scale and incubated at 23 °C and 750 rpm in a thermomixer for 5 h. Uniformly labeled HttEx1 was obtained by substituting the standard amino acid mix with 3 mg/mL [^15^N,^13^C]-labeled ISOGRO^®^ [53] (an algal extract lacking four amino acids: Asn, Cys, Gln and Trp) and additionally supplying [^15^N,^13^C]-labeled Asn, Cys, Gln and Trp (1 mM each, CortecNet, Les Ulis, France). To enable the labeling of glutamates, potassium glutamate was substituted by potassium acetate. The optimal potassium acetate concentration was determined by testing a range of concentrations.

Selective labeling of one or more amino acids, e.g., only Phe, was achieved by removing the respective amino acids from the standard amino acid mix and replacing them with 1 mM of the labeled amino acid, e.g., [^15^N,^13^C]-Phe (CortecNet, Les Ulis, France) [54,55]. The same approach was applied to remove the natural abundance peaks of Gln and Pro to improve the quality of the ^13^C-HSQC spectra. Gln and Pro were removed from the standard amino acid mix and replaced by deuterated Gln and Pro (Eurisotop, Saint-Aubin, France) [52]. All HttEx1 constructs were purified as previously described [20,52].

#### 2.3.5. Site-Specific Isotopic Labeling in CF Expression Systems

To produce site-specifically labeled samples 10 µM of [^15^N,^13^C]-Gln or [^15^N,^13^C]-Pro suppressor tRNA_CUA_ were added to the CF reaction. To improve the quality of the spectra by removing the natural abundance peaks of Gln and Pro, all Gln and all Pro (excluding the suppressed residue) were deuterated. See section on selective labeling for details. SSIL HttEx1 samples for NMR studies were produced at 5−10 mL scale and incubated at 23 °C and 750 rpm in a thermomixer for 5 h, before being purified as previously described [20,52].

#### 2.3.6. Addition of EF-Tu to CF Expression and CF Suppression Reactions

To evaluate the effect of additional EF-Tu on a standard CF reaction expressing H16-sfGFP, the reaction was titrated with increasing amounts of activated EF-Tu (see above) (0, 1, 2.5, 5, 10 and 20 µM). Similarly, the effect of EF-Tu on a CF suppression reaction was investigated. To this end, activated EF-Tu and equal amounts of aa-tRNA_CUA_ (0, 5, 10 or 20 µM) or preformed equimolar complexes of active EF-Tu and aa-tRNA_CUA_ (0, 5, 10 or 20 µM) were added to the CF reactions. The time-course of H16 protein synthesis was monitored using a fluorescence read-out as described in 2.3.3.

### 2.4. EF-Tu:aa-tRNA_CUA_ Stability Assay

In order to determine if the addition of active EF-Tu and formation of the ternary EF-Tu:tRNA complex protected the ester bond between the amino acid and the tRNA from hydrolysis, 2 µM aa-tRNA_CUA_ was incubated with 4 µM activated EF-Tu in 55 mM HEPES pH 7.5 at 30 °C. As a reference, 2 µM aa-tRNA_CUA_ was incubated without any EF-Tu under the same conditions. Samples were taken and subsequently analyzed by urea-PAGE (6.5% acrylamide 19:1, 8 M urea, 100 mM sodium acetate pH 5.2) [56].

### 2.5. NMR Experiments

NMR experiments were performed as previously described [20,25,52]. Briefly, all NMR samples contained final concentrations of 10% D_2_O and 0.5 mM 4,4-dimethyl-4-silapentane-1-sulfonic acid (DSS). Experiments were performed at 293 K on a Bruker Avance III spectrometer (Bruker Biospin, Wissembourg, France) equipped with a cryogenic triple resonance probe and Z-gradient coil, operating at a ^1^H frequency of 700 or 800 MHz. ^15^N-HSQC and/or ^13^C-HSQC were acquired for the respective samples. Spectra acquisition parameters were chosen depending on the sample concentration and the magnetic field. All spectra were processed with TopSpin v3.5 (Bruker Biospin, Wissembourg, France) and analyzed using CCPN-Analysis software v2.4 [57]. Chemical shifts were referenced with respect to the H_2_O signal relative to DSS using the ^1^H/X frequency ratio of the zero point according to Markley et al. [58].

## 3. Results

### 3.1. An Optimal Huntingtin Exon-1 Construct for CF Expression

As aggregation-prone protein, huntingtin exon-1 (HttEx1) is normally produced fused to soluble proteins such as thioredoxin (Trx), maltose binding protein (MBP) or glutathione S-transferase (GST) [7,8,9,23]. Here, we selected a fluorescent fusion protein in order to facilitate the multiple optimization steps required for CF production. By placing the globular protein at the C-terminus, complete synthesis of the target protein could be easily monitored as truncation products arising from incomplete suppression (see below) were not fluorescent. Moreover, this design preserves the topology of full-length huntingtin, which has a globular HEAT-repeat at the C-terminus of HttEx1 [3]. Superfolder green fluorescent protein (sfGFP) [59] was selected as fusion protein based on its faster folding kinetics, which reduces the potential contact of the aggregation-prone HttEx1 with the exposed hydrophobic core of sfGFP during its folding. Between HttEx1 and sfGFP, a 3C protease site was introduced to enable access to untagged HttEx1 for biophysical studies. Due to the sequence requirements of 3C protease and the employed cloning strategy, eight non-native amino acids (EASLEVLFQ) remain after cleavage. Moreover, a His-tag was incorporated after the sfGFP for affinity purification of the complete product in a single step. The final construct (Figure 1b) was cloned into a pIVEX 2.3d plasmid for CF expression. The same architecture was used for a sub-pathological and a pathological version of HttEx1, containing 16 (H16) and 46 (H46) consecutive glutamines, respectively, which we used in our structural studies. Importantly, similar yields were obtained for a construct containing a Trx at the N-terminus (data not shown). Although this construct was not used in our studies, it could be useful when producing HttEx1 with longer poly-Q tracts [8].

### 3.2. Optimization of CF Expression Conditions

**Figure 2:**
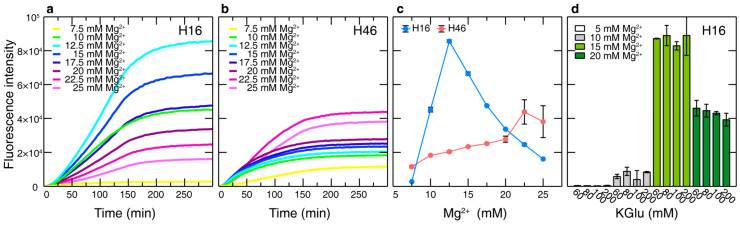
Mg^2+^ and K^+^ titrations. The fusion to sfGFP allows the real-time fluorescence readout of the Mg^2+^ titration of (**a**) H16 and (**b**) H46. In addition to determining the optimal Mg^2+^ concentration, the time-resolved data show when the maximum expression level has been reached. This enables the adjustment of the incubation time of large-scale expressions for each lysate. (**c**) Plot comparing the dependence of H16 and H46 expression on Mg^2+^ for a specific lysate. (**d**) Titration of H16 with different Mg^2+^ and K^+^ concentrations. While varying the Mg^2+^ concentration strongly affected the expression, it was less sensitive towards variations of the K^+^ concentration.

The lysate production and the CF expression protocols used in this study were based on those developed in Otting’s lab [40,51]. Note that we use an induced lysate in which the T7 polymerase has been overexpressed. Details of the lysate production and the batch CF protocol used here have been previously reported [25]. As in all CF protocols, almost all parameters can be optimized in order to improve production yields [60]. In our hands and in line with most protocols, the Mg^2+^ concentration is the most critical parameter. Figure 2 displays typical production curves for H16 and H46 as a function of the Mg^2+^ concentration. For H16 large differences in production are observed in the 7.5 to 25 mM range, with a narrow peak at 12.5 mM. For this specific lysate, H46 displays an overall lower protein production and a less acute Mg^2+^ concentration dependence than H16 with a maximum yield at higher Mg^2+^ concentration (22.5 mM). This last trend was observed for all 15 lysate batches produced along the study. For H16, the optimal Mg^2+^ concentration was 12.5 mM in 14 batches, while in one batch the optimal value slightly deviated 15 mM as optimal Mg^2+^ concentration. Conversely, a broad range of optimal Mg^2+^ concentration, ranging from 10 to 20 mM, was obtained for the production of H46.

In addition to determining the optimal Mg^2+^ concentration for a given lysate and construct, it is customary to optimize the K^+^ concentration [61]. Figure 2d displays the total production of H16 when simultaneously varying the Mg^2+^ (5–20 mM, magnesium acetate, Mg(OAc)_2_) and K^+^ (5–200 mM potassium glutamate, KGlu) concentrations. While changes in the Mg^2+^ concentration have a strong effect on the production, yields are virtually not affected by the change in the amount of K^+^ in the reaction. Moreover, this lack of sensitivity on the K^+^ concentration does not depend on the lysate batch. Similar results have been observed for H46 (data not shown). Although the final yield is not affected by the K^+^ concentration, its increase slows down protein synthesis (data not shown). Taking into account that fast production is important for site-specific labeling (see below), 100 mM of KGlu was systematically used in all experiments.

### 3.3. Optimization of the Glutamine Concentration

**Figure 3:**
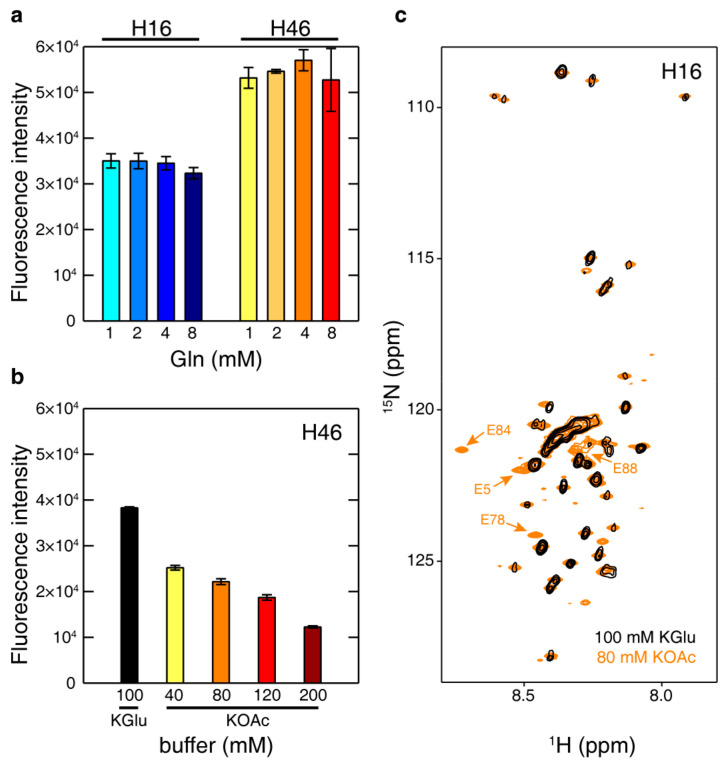
Optimization of cell-free (CF) expression conditions to enable the uniform isotopic labeling of HttEx1. (**a**) Titration of CF reactions of H16 and H46 with increasing concentrations of glutamine. (**b**) KGlu was substituted by KOAc to enable the isotopic enrichment of glutamate residues. Different concentrations of KOAc were tested to improve H46 expression yields. (**c**) Overlay of ^15^N-HSQC spectra of H16 produced in a KGlu buffered CF reaction (black) and H16 produced in a KOAc buffered system (orange). The additional peaks (indicated with an arrow) arise from glutamic acid residues in the construct (H16 and linker) that were not visible in the sample produced with KGlu. The remaining peaks correspond to glutamic acid residues from sfGFP.

The most interesting feature of HttEx1 is the presence of a tract of glutamines of variable length. Standard CF protocols use each amino acid at 1 mM concentration. However, this concentration can be increased if the protein produced is rich in one specific amino acid. For instance, an additional amount of alanine is introduced in the mixture when T7 polymerase is also synthesized in the same CF reaction [51]. In this context, we explored how the total concentration of glutamine present in the CF reaction affected the expression yield of HttEx1. Therefore, multiple small-scale batch CF reactions with increasing amounts of glutamine in the reaction mixture were performed for H16 and H46 (Figure 3a). These experiments clearly demonstrate that the protein production is not affected by the glutamine concentration. Interestingly, this is observed for both constructs, which differ by the presence of 30 glutamine residues.

### 3.4. Uniform Isotopic Labeling of Huntingtin

NMR sequential assignment requires the uniform ^13^C and ^15^N isotopic labeling of samples. Although uniform labeling is straightforward in CF expression systems when using a mixture of isotopically labeled amino acids, this is an expensive approach. Commercial algal extracts represent an efficient and economical alternative for labeling proteins in CF as they can be purchased with all isotopic (^13^C, ^15^N and ^2^H) combinations of amino acids and can be added directly to CF reactions. Importantly, algal extracts are depleted in four amino acids: Gln, Asn, Cys and Trp, which must be additionally supplied. Using the protocol for CF sample production mentioned above, Glu residues do not appear in NMR spectra (see below). This is due to the high concentration, 100 mM, of potassium glutamate (KGlu) present in the standard reaction mix, which exceeds the amount of this amino acid present in labeled amino acid mixtures or algal extracts. Furthermore, scrambling arising from the metabolic conversion of Glu into Gln induces an isotopic dilution and a systematic decrease of Gln peak intensities [29,62,63]. To solve this issue, we evaluated the performance of our CF reactions when potassium acetate (KOAc) was used instead of KGlu. We tested increasing amounts of KOAc for several H16 CF syntheses using ISOGRO^®^ algal extract (Figure 3b). Our data indicate that H16 can be synthesized when using KOAc; however, the production is 34% less efficient in 40 mM KOAc than it is in 100 mM KGlu. Despite this decrease, the combination of algal extract with KOAc is an excellent and cost-effective alternative to produce uniformly isotopically labeled samples for sequential NMR assignment. This is corroborated in Figure 3c where the ^15^N-HSQC spectra of H16 produced in algal extract using KGlu or KOAc are overlaid. Note that peaks corresponding to E5, E78, E84 and E88, indicated with an arrow, are only visible in the sample produced in KOAc.

### 3.5. HttEx1 Production Variability Across Different Lysates

**Figure 4:**
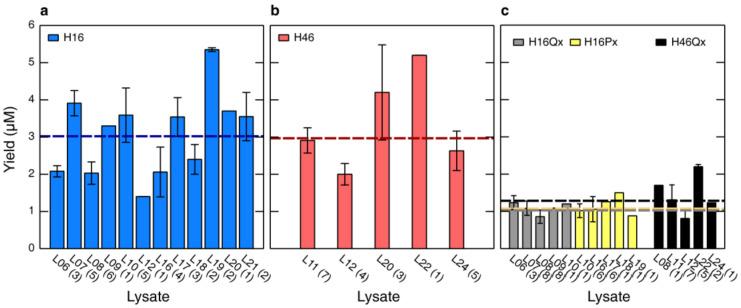
HttEx1 production variability across different lysates. Average CF yields obtained with different lysate batches for (**a**) H16 and (**b**) H46. The mean yield across all samples is shown as a dashed line. (**c**) Average yields obtained for SSIL samples of H16 Gln (gray), H16 Pro (yellow) and H46 Gln (black). The mean yield across all samples is shown as gray, yellow and black dashed lines, respectively. In all panels, the lysate batch and the number of samples prepared in the respective lysate are given (e.g., L06 (3)).

The structural characterization of HttEx1 has required the preparation of multiple samples with different isotopic labeling schemes. The large quantity of samples of the same protein prepared using different lysates enables the evaluation of the robustness of our procedures. Figure 4 displays the average yield of H16 and H46 for 15 different lysates. A total of 55 CF reactions (35 for H16 and 20 for H46) are reported. Note that in addition to the lysate batches, the total volume and the operators were different for the individual productions [64]. For H16 and H46 the final average concentrations reached at the end of the reaction were 3.02 ± 1.1 and 2.97 ± 1.0 μM, respectively. This similarity suggests that the number of glutamines present in the protein is not impacting the efficiency of the HttEx1 production. Some variability was observed between lysates. Lysates L07, L19 and L22 seem to be systematically more efficient than the average, while L06, L08 and L12 seem to be less efficient. The same trend was observed for lysates that were used to produce both proteins, L12 and L20. Interestingly, for these two lysates H46 was produced more efficiently than H16. Unfortunately, we do not have enough statistics to derive solid conclusions regarding the differential expression of H16 and H46 and the parameters influencing it.

We have attempted to connect yields displayed in Figure 4a,b with the specificities of the individual lysate productions. Although no clear correlation could be established, lower-yield lysates seem to correspond to those for which problems with the cell lysis occurred. In this step, cell pellets that are not well resuspended can block the French-press and their removal will lead to the dilution of the lysate. In addition, the cells and the lysate are exposed to higher temperatures. This observation substantiates the previous observations underlining the importance of bacterial lysis to achieve highly productive lysates [65,66].

### 3.6. CF Synthesis as a Flexible Platform for Tailored Isotopically Labeled HttEx1 Samples

**Figure 5:**
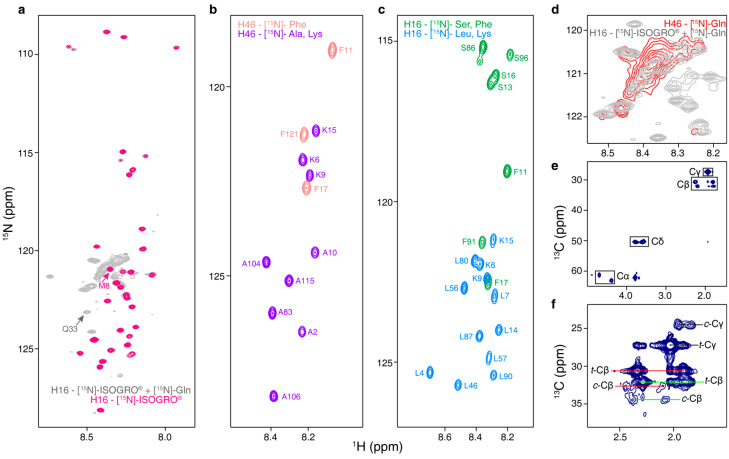
Isotopic labeling strategies for the NMR investigation of HttEx1. (**a**) Overlay of the ^15^N-HSQC spectra of H16 samples expressed using [^15^N]-ISOGRO^®^ in the absence (pink) or presence (gray) of [^15^N]-Gln, as shown previously in [25]. (**b,c**) ^15^N-HSQC spectra of selectively labeled H46 (^15^N-Phe and [^15^N]-Ala/[^15^N]-Lys) and H16 (^15^N-Ser/^15^N-Phe and [^15^N]-Leu/[^15^N]-Lys) samples, respectively. (**d**) Zoom of the Gln region of ^15^N-HSQC spectra of fully labeled H16 and exclusively [^15^N]-Gln -labeled H46. (**e**) ^13^C-HSQC spectra of an H46 sample exclusively labeled with [^15^N,^13^C]-Pro. (**f**) Zoom of the ^13^C-HSQC from panel (**e**), highlighting the correlations of the *cis* and *trans* conformations of C_γ_-H_γ_ (black) and of the two proline families observed for C_β_-H_β_, Pro-*Pro* (red) and Pro-*Xaa* (green).

The compositional bias of HttEx1 causes a severe signal overlap in NMR spectra [10,11,15,20,25]. The efficient and robust CF expression protocols available and the straightforward modification of the composition of the reaction mixture offer interesting isotopic labeling possibilities that enable easier NMR investigations. This flexibility is exemplified in Figure 5; Figure 5a compares the ^15^N-HSQC spectra of fully labeled H16, using [^15^N]-labeled algal extract, 80 mM KOAc and 4 mM of ^15^N-Gln, with a sample produced with [^15^N]-labeled algal extract without isotopically-labeled Gln and buffered with KGlu. The overlap of the peaks belonging to N17 and the PRR is perfect while the broad density corresponding to the poly-Q region is missing in the non-labeled Gln sample. Glu residues are also missing due to the use of non-labeled KGlu. The comparison of these two spectra offers two advantages. First, we can identify those Gln peaks that lie outside of the broad density such as Q33. Second, residues, such as M8 whose peak is hidden in the broad Gln region, can be assigned.

To further simplify the spectra, only one or a few amino acids can be isotopically labeled (Figure 5b,c). Note that the low concentrations reached for pathological HttEx1 constructs as well as their aggregation propensity preclude measuring traditional 3D spectra for sequential assignment and thus the access to structural information of HttEx1. Under these circumstances, the identification of amino acid types can be useful to cross validate the traditional assignment performed on less aggregation-prone HttEx1 variants. This is shown in Figure 5b for two H46 samples, labeled with either ^15^N-Phe or ^15^N-Ala/^15^N-Lys. Analogous samples of H16 are shown in Figure 5c, labeling either ^15^N-Ser/^15^N-Phe or ^15^N-Leu/^15^N-Lys. This strategy also allows the detailed investigation of structural phenomena. In a previous study, we identified residues preceding the poly-Q tract, ^14^LKSF^17^, as helical promoters [20]. By exclusively monitoring these residues (Figure 5c), perturbations of this property induced by mutations or experimental conditions can be investigated.

For proteins containing low-complexity regions, the exclusive labeling of the enriched amino acids can be highly valuable to monitor the differences in their chemical environments through their chemical shifts. In Figure 5d, only the backbone ^1^H-^15^N Gln signals of H46 are observed and shown in comparison with the spectrum of fully labeled H16. The Gln density of both spectra share similar features, like the modest dispersion of the peaks for residues closer to the N17 region (upfield part), indicating more helical conformations and the poor dispersion of the peaks belonging to residues close to the PRR (downfield part), which preferentially adopt random coil conformations [20,25]. However, the intensity increase observed in the middle section of the broad glutamine peak in H46 suggests that the majority of the additional glutamines in this construct adopt lowly populated helical conformations, rather than random coil conformations. The latter would result in an intensity increase at the bottom part of the glutamine peak. Therefore, a qualitative perspective of the structural perturbations occurring at the homorepeat beyond the pathological threshold can be derived.

Downstream of the poly-Q, HttEx1 contains a PRR with 31 prolines, including two poly-P tracts with 11 and 10 prolines, respectively (Figure 1a). The side chain of proline is covalently bound to the backbone nitrogen atom, forming a pyrrolidine ring that provides this amino acid with special features. The most relevant of them is the relatively high population of the *cis* conformation of the *Xaa*-Pro amide bond. This property has been the target of many structural and biophysical studies [67,68,69,70]. Figure 5e displays the ^13^C-HSQC of a H46 sample where Pro was the only isotopically labeled (^13^C and ^15^N) amino acid introduced. The most relevant aspect is the reduced number of peaks, indicating a limited number of Pro classes in HttEx1. In fact, two families of prolines can be identified, those followed by another Pro (Pro-*Pro*) and those followed by any of the other amino acids (Pro-*Xaa*). These two families give rise to two peaks for C_α_-H_α_ and C_β_-H_β_, while they present the same frequency for C_γ_-H_γ_ and C_δ_-H_δ_. In the upper part of the spectrum, minor peaks, corresponding to the *cis* conformation, can be observed (Figure 5e,f). Notice that only C_β_-H_β_ and C_γ_-H_γ_ correlations are sensitive to the presence of *cis* conformations. Interestingly, this spectrum is equivalent to that measured for H16 [52]. With the intensities of the C_β_-H_β_ and C_γ_-H_γ_ peaks displayed in Figure 5f, we calculated the average population of *cis* conformations for all prolines in H46 (4.0%) and the average population of this conformer for prolines belonging to the Pro-*Pro* (2.6%) and Pro-*Xaa* (6.5%) families. Interestingly, these values are in excellent agreement with those derived for H16, 4.0%, 3.0% and 7.4%, respectively [52]. This suggests that the increase in the poly-Q length does not affect the conformational behavior of the PRR.

### 3.7. Glutamine and Proline Site-Specific Isotopic Labeling

Even in its non-pathogenic form, the homorepeat in HttEx1 contains a large number of glutamines [3,71]. Together with its intrinsic disorder, this chemical uniformity results in severe signal overlap in NMR spectra, which limits the application of traditional 3D-NMR for assignment (see above). Thus, in order to enable the study of glutamine homorepeats independently of the poly-Q length, we developed a site-specific isotopic labeling (SSIL) strategy [25]. Since HttEx1 also contains a PRR that encompasses two poly-P tracts and plays an important role in modulating huntingtin aggregation and fibril formation [13,16,17,72,73], we expanded the SSIL approach to proline [52].

**Figure 6:**
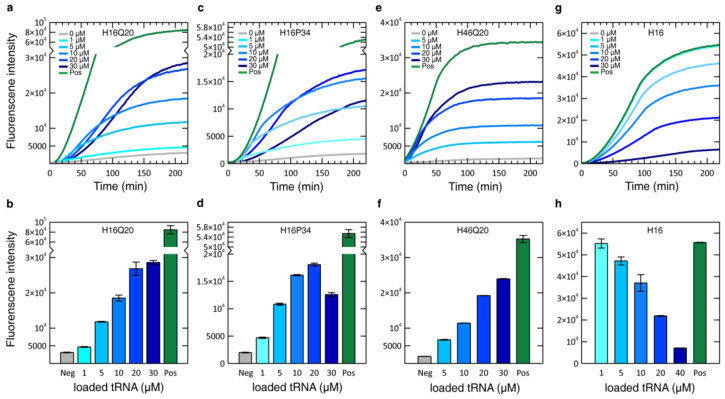
Optimization of CF suppression reactions. The kinetics and final expression levels when using increasing concentrations of aa-tRNA_CUA_ are shown. Time-resolved fluorescence and endpoint data of a CF suppression reaction expressing H16 with an amber codon at position Q20 using Gln-tRNA_CUA_ (**a**,**b**) or at position P34 using Pro-tRNA_CUA_ (**c**,**d**). Time-resolved fluorescence and endpoint data (**e**,**f**) of a titration of H46Q20 with an amber codon at position Q20 using Gln-tRNA_CUA_. Time-resolved fluorescence (**g**) and endpoint data (**h**) of a titration of H16 without any amber codon with loaded Pro-tRNA_CUA_. Neg indicates negative controls of the corresponding amber plasmid without added aa-tRNA_CUA_. Pos indicates positive controls using a plasmid without an amber codon and without added aa-tRNA_CUA_.

By combining CF protein expression with nonsense suppression [42], individual glutamine or proline residues in HttEx1 can be isotopically labeled [25,52]. Since the aminoacyl tRNA synthetases (aaRSs) responsible for charging tRNAs with their cognate amino acids cannot distinguish between isotopologues, the nonsense suppressor tRNA (tRNA_CUA_) has to be loaded with the labeled amino acid externally, yielding aa-tRNA_CUA_ that is subsequently added to the CF reaction. Titration experiments using H16-sfGFP bearing an amber stop codon instead of either a glutamine or proline codon showed a concentration-dependent increase in fluorescence that reached its maximum at 20 µM aa-tRNA_CUA_ and then decreased for larger concentrations, as shown in Figure 6 for Q20 and P34. The origin of this decrease at large aa-tRNA_CUA_ was investigated by monitoring the protein synthesis kinetics (Figure 6a,c). While the translation is accelerated notably upon the addition of increasing amounts of aa-tRNA_CUA_ up to a concentration of 10 µM aa-tRNA_CUA_, it started to slow down at higher aa-tRNA_CUA_ concentrations. The combination of the slower kinetics and the limited reaction time of a batch CF system causes an overall decrease of the final yield at high suppressor concentrations. Notice that at 20 µM of aa-tRNA, the final yield is larger than at 10 µM; however, the increase is less than two-fold. For that reason, 10 µM was considered the optimal concentration for large-scale NMR sample production. Similar observations were made with H46 with an amber codon at position Q20, although a plateau was not reached in that case (Figure 6f). The expression kinetics are faster than those observed for H16Q20 and H16P34; however, this may be a lysate-dependent effect (Figure 6a,c,e).

In order to understand the suppressor concentration-dependent kinetics, we investigated the effect of Pro-tRNA_CUA_ on the expression of an H16 construct without any amber codon (Figure 6g,h). Like in the previous experiments, with increasing concentrations of Pro-tRNA_CUA_, the final yield of the CF reaction decreased, and the translation kinetics slowed down visibly, indicating that the presence of large concentrations of aa-tRNA_CUA_ had an effect on the CF reaction. We speculate that the addition of a large quantity of aa-tRNA_CUA_ is perturbing the subtle equilibrium of all biomolecules involved in the translation mechanism, such as ribosomes, aaRSs and tRNAs, and several initiation, elongation and release factors [74], reducing its efficiency. Even though the exact concentrations and ratios of these components are not known, and vary between lysates, efforts have been made to investigate the composition of S30 lysate and potential factors that are limiting translation in CF systems [75,76]. While current S30 lysates seem to present optimal ratios between the all components necessary for an ordinary translation reaction [75], the addition of aa-tRNA_CUA_ seems to disturb this balance. Based on the number of tRNA molecules present in each cell at specific growth rates and doubling times [77], we estimated the total tRNA concentration of our lysate to be ~10 µM. Moreover, we supply our CF reaction with additional commercially available *E. coli* tRNAs (0.175 mg/mL ≈ 7 µM). Therefore, in suppression experiments, between one third and one half of the lysate’s total tRNA is added in the form of aa-tRNA_CUA_. This large amount of the same type of tRNA competes for binding to elongation factor EF-Tu. Since the amber codon is present only once per mRNA transcript, the EF-Tu/aa-tRNA_CUA_ complex has fewer opportunities to release its charge, hence preventing the release of EF-Tu to bind to another molecule of aa-tRNA, severely slowing down the translation process.

### 3.8. EF-Tu as an Additive for CF Protein Production

The addition of EF-Tu to CF reactions has been previously proposed as a strategy to increase protein production when applying the tRNA suppression strategy [78]. In our case, the addition of EF-Tu to CF reactions had two main purposes. First, we wanted to evaluate the capacity of this protein to increase the stability of the ester bond between the tRNA_CUA_ and loaded amino acid. Second, we aimed at balancing the concentrations between EF-Tu and aa-tRNA_CUA_ in order to improve the protein synthesis yields under suppression conditions.

**Figure 7:**
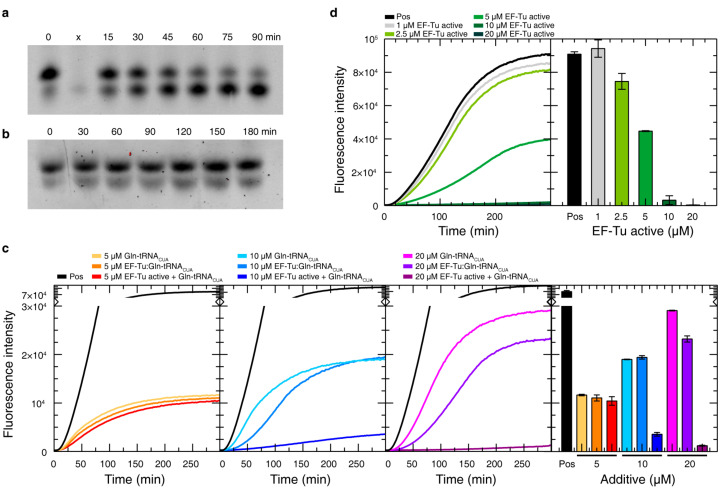
Effect of EF-Tu on Gln-tRNA_CUA_ stability and CF HttEx1 synthesis. Deacylation assay of Gln-tRNA_CUA_ in the absence (**a**) and in the presence (**b**) of a 2x excess active EF-Tu. (**c**) Time-resolved fluorescence and endpoint fluorescence for CF suppression reactions of H16 with a stop codon at position Q20 when titrating with increasing concentrations of Gln-tRNA_CUA_ alone, a preformed complex of Gln-tRNA_CUA_ and active EF-Tu (EF-Tu:Gln-tRNA_CUA_) or EF-Tu active + Gln-tRNA_CUA_. (**d**) Time-resolved fluorescence and endpoint fluorescence for a standard CF reaction of H16 without any amber stop codon when titrated with increasing concentrations of active EF-Tu. Pos indicates positive controls using a plasmid without an amber codon and no additives. The original images corresponding to (**a**) and (**b**) are shown in Figure S1.

At neutral pH and 30 °C, the ester bond connecting the tRNA_CUA_ with the amino acid is labile to hydrolysis. We monitored the deacylation process with PAGE-gels in which we observed the two bands corresponding to the tRNA_CUA_ in its free form (bottom band) and when acetylated (top band) (Figure 7a). According to this experiment, around 90% of the Gln-tRNA_CUA_ is deacetylated in 90 min, which corresponds to the end point of CF protein synthesis under suppression conditions. Interestingly, in the presence of EF-Tu in a 1:2 ratio, the tRNA_CUA_ remains aminoacylated for more than 90 min, demonstrating the capacity of the protein to protect the ester bond (Figure 7b).

After demonstrating that EF-Tu can efficiently protect Gln-tRNA_CUA_ from deacylation, we tested whether the addition of the preformed complex to the CF reaction had a positive effect on the synthesis of H16 suppression samples (Figure 7c). While the addition of 5–10 µM EF-Tu:Gln-tRNA_CUA_ did neither improve nor worsen the overall yield of the synthesis when compared with the addition of naked Gln-tRNA_CUA_, the addition of 20 µM complex resulted in a lower yield. The analysis of the time-resolved fluorescence intensity provides insights into the reasons of this decrease (Figure 7c). The addition of the complex slows down the overall reaction kinetics by increasing the lag-phase. Interestingly, the growth rate is faster in the presence of the EF-Tu:Gln-tRNA_CUA_ complex, although not enough to compensate the slower initiation time due to the limited reaction time available in CF batch reactions. In line with these observations, the separate addition of equimolar concentrations of free activated EF-Tu and Gln-tRNA_CUA_ was shown to decrease the final yield of suppressed CF reactions, which is especially severe at 10 and 20 µM.

The effect of increasing amounts of EF-Tu on the CF production of non-suppressed H16 was also evaluated (Figure 7d). While low concentrations (1 or 2.5 µM EF-Tu) did not affect the yield of the synthesis, 5 µM EF-Tu reduced the yield by 50%, and 10 and 20 µM EF-Tu led to complete inactivation of the protein synthesis. Again, this observation suggests that disturbing the balance of the relative quantities of biomolecules involved in protein translation has a strong impact on the process and turns out to be very difficult to compensate.

### 3.9. Reproducibility of SSIL in CF Reactions

With a CF reaction optimized for SSIL, the reaction size was scaled up (5–10 mL) to prepare HttEx1 samples for NMR analysis. Owing to the fact that for each investigated glutamine or proline a separate sample had to be produced, a large number of similar samples were prepared, requiring several batches of lysate. This allowed us to compare the expression yields and thus to evaluate the robustness of our lysate preparation protocol and CF suppression reaction. Figure 4c displays the yields for the 52 SSIL samples produced for H16 and H46 along the study using 13 different lysates. The average concentration at the end of the CF reaction was 1.02 ± 0.23 µM and 1.07 ± 0.28 µM for H16 glutamine and H16 proline suppression, respectively. This represents 34% of the yield of the same protein under non-suppression conditions (3.02 ± 1.1 μM, see Figure 4a), which is comparable to previous studies [79,80]. Interestingly, both amino acids tested provided equivalent averaged yields, indicating that the suppression process is not strongly influenced by the chemical properties of the amino acid and the sequence of the tRNA_CUA_. In a previous study, we also observed that the percentage of loaded tRNA_CUA_ does not strongly influence the final yield of the reaction [52]. For H46 glutamine suppression, the average concentration at the end of the CF reaction was slightly higher with 1.28 ± 0.52 µM. In comparison with the average concentration of non-suppressed H46 (2.97 ± 1.0 µM), this corresponds to 43%. These observations suggest that the length of the homorepeat has no impact on the production of SSIL samples.

Interestingly, suppressed samples of H16 display a reduced yield variability between different lysates compared with that observed for non-suppressed ones. In that sense, lysates that were less efficient with non-suppressed samples, such as L06 or L16, did not show lower-than-average levels of suppression. In contrast, the variability in yield of H46 suppression samples seems to be more closely correlated to the observed variability of non-suppressed H46 samples expressed in different lysates. For instance, lysate L22 displays the highest yields in both suppressed and non-suppressed samples. These observations suggest that the performance of the lysate, although not critical, can be a relevant parameter for SSIL sample production, although a larger number of samples should be produced to assess its exact contribution. Overall, these observations indicate that the CF production of SSIL samples is a robust process that is not sensitive to most of the experimental parameters.

### 3.10. NMR on Site-Specific Isotopically Labeled Samples

**Figure 8:**
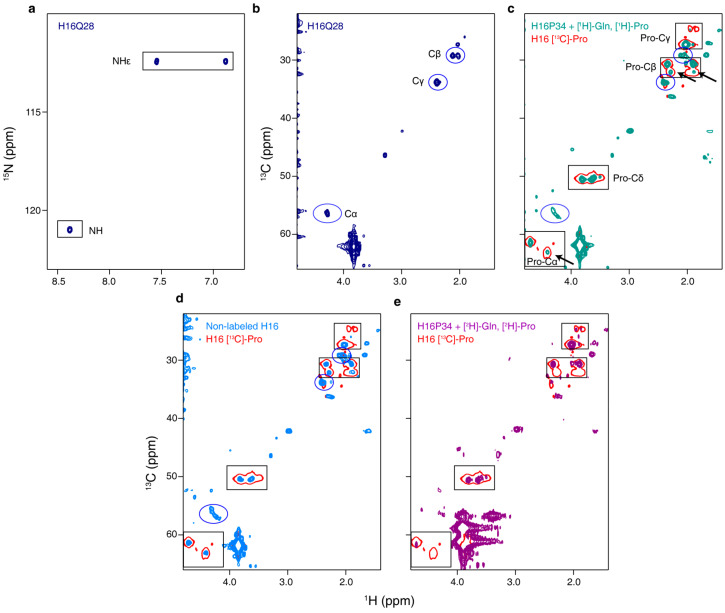
Site-specific isotopic labeling of H16. (**a**,**b**) ^15^N and ^13^C-HSQC spectra of site-specifically [^15^N,^13^C]-labeled Q28 of H16. (**c**) ^13^C-HSQC of H16 P34 (dark green) expressed in a CF reaction using non-deuterated Pro and Gln. The spectrum of a H16 sample with all Pro ^13^C-labeled (H16 [^13^C]-Pro) is shown in red. Blue circles and black arrows indicate the presence of natural abundance CH signals arising from Gln and Pro, respectively. (**d**) Overlay of the ^13^C-HSQC spectra of H16 [^13^C]-Pro (red) and a non-labeled H16 (blue) sample. (**e**) Overlay of the ^13^C-HSQC spectra of H16 [^13^C]-Pro (red) and H16P34 expressed in a CF reaction in the presence of deuterated Pro and Gln (purple).

The utility of SSIL to simplify the NMR analysis is exemplified in Figure 8 where panels a and b show the ^15^N- and ^13^C-HSQC spectra of a sample where Q28 of H16 was labeled site-specifically. In both types of spectra, all nuclei present well-isolated peaks. In the ^15^N-HSQC, the backbone ^15^N-H and side chain ^15^N-H_2_ signals can be identified with ease (Figure 8a). Similarly, the ^13^C-HSQC spectrum in panel b shows peaks corresponding to C^α^-H^α^, C^β^-H^β^ and C^γ^-H^γ^. As expected for a partially disordered homorepeat, the two H^β^ chemical shifts of the Gln are distinct, while the two H^γ^ ones are not [20,81].

As a second application, we extended our SSIL strategy to proline. As mentioned above, this amino acid is of particular interest due to the conformational properties of the *Xaa*-Pro amide bond. In order to investigate the *cis/trans* equilibrium of prolines in the PRR of H16, we prepared SSIL samples for several prolines within and outside of the poly-P tract. An early ^13^C-HSQC spectrum of site-specifically labeled P34 of H16 is shown in Figure 8c. Unfortunately, the presence of a large number of glutamine and proline residues in HttEx1 and the reduced dispersion of their chemical shifts result in the appearance of natural abundance ^13^C-H signals that hinder the unambiguous assignment. Despite only labeling P34, which belongs to the Pro-*Pro* family, C^α^-H^α^ and C^β^-H^β^ signals from other prolines (red densities) are visible in the ^13^C-HSQC. This is particularly clear, since signals corresponding to the Pro-*Xaa* family were also observed in the spectrum (Figure 8c, black arrows). Moreover, ^13^C-H correlations arising from glutamines (blue circles) are detected as well. The fact that the observed signals were indeed arising due to natural abundance was confirmed by recording a ^13^C-HSQC of a non-isotopically enriched H16 sample (Figure 8d), which displays the same signals as observed in Figure 8c. Importantly, the spectral contamination from prolines other than the suppressed one precludes the quantitative determination of peak intensities and, consequently, the precise site-specific determination of the relative *cis/trans* population. In order to suppress the appearance of natural abundance signals, SSIL samples need to be prepared in presence of deuterated Gln and Pro in the CF reaction. Then, clean spectra of the desired proline are obtained without the presence of additional peaks. This is exemplified in the ^13^C-HSQC spectrum shown in Figure 8e, where a single C^α^-H^α^ peak and two C^β^-H^β^ peaks are observed at the frequencies corresponding to the Pro-*Pro* family.

## 4. Discussion

The investigation of proteins with low-complexity sequences represents a challenge for traditional structural biology approaches [27,44]. Due to their inherent flexibility, NMR is the most suited technique for these studies. However, the uniform isotopic labeling of low-complexity sequences leads to highly congested NMR spectra that hamper sequential assignment. Here, we demonstrate that CF protein synthesis is an extremely well-suited platform for the structural investigation of LCRs by NMR. To demonstrate this point, we have studied HttEx1, arguably the most notorious example of homorepeat proteins [82,83,84] due to the direct link between the size of the poly-Q and the appearance of neurodegenerative HD.

With our standard lysate production protocol, we obtain robust batches for the efficient synthesis of HttEx1 fused to sfGFP. Importantly, the final yields (≈3 μM) are not affected by the extension of the poly-Q tract, giving access to sub-pathological and pathological versions of the protein. Although we have only attempted the production of H46, we speculate that other pathological HttEx1 constructs with longer poly-Q tracts could be obtained using the same protocol and with similar yields. However, potential problems in the purification step due to the enhanced aggregation propensity could hamper the production of viable NMR samples for these constructs. In fact, our H46 NMR samples have systematically lower concentrations than those of H16, despite the yields at the end of the CF being virtually equivalent. This suggests that protein purification but not CF production is the critical step for the preparation of pathological HttEx1 NMR samples.

In our studies, we have privileged the batch mode over the continuous-exchange mode, which allows longer reaction times and results in higher yields [28,85]. Batch mode requires smaller volumes and lower amounts of the components, making it more cost efficient for isotopic labeling. Interestingly, in our attempts to produce H46 using continuous exchange, fluctuations in the fluorescence signal were observed after few hours of reaction (results not shown). We interpreted this behavior to be due to the formation of aggregates and/or liquid droplets of the protein in the CF mixture. Note that liquid–liquid phase separation has been observed in vitro and in vivo for HttEx1 constructs with different poly-Q lengths [12].

CF has been widely used for the production of proteins for structural studies by NMR [36,37]. Our work now demonstrates that it is especially suited to tackle proteins containing low-complexity regions. The flexibility in the composition of the amino acid mixtures allows the specific labeling (or absence of labeling) of individual amino acids, enabling focusing on specific structural features. For instance, the specific labeling of Gln in H46 suggests that the majority of residues of the poly-Q display partially helical conformations, in line with previous circular dichroism experiments of pathological HttEx1 [86]. The specific labeling of Pro in H46 has enabled the quantification of the percentage of *cis* conformers in the PRR, demonstrating that the length of the poly-Q tract does not modify the conformational preferences in that region. Moreover, the addition of deuterated amino acids has also been key to avoid natural abundance peaks in suppression samples. The main limitation of amino acid specific labeling is the presence of scrambling processes, where one amino acid is enzymatically converted into another. Although most of these processes are severely compromised in CF preparations, some of them, such as the Gln/Glu and Asn/Asp scrambling, are still active in standard lysates [29,62,63]. This feature is especially relevant for the study of HttEx1 where the Gln labeling is partially diluted by the presence of unlabeled Gln originating from KGlu, which is the main component of the CF mixture. This problem can be efficiently overcome by the use of KOAc as the basic component of the CF buffer [29]. Moreover, this replacement allows the isotopic labeling of Glu, which is key for sequential assignment and might be important for interaction experiments by NMR. However, this modification also impacts the yield of the expression, which decreases around one third for HttEx1. Note that similar decreases in protein production have also been reported when glutamine synthetase inhibitors are added to the CF reaction [62]. Another option to prevent scrambling is the use of the PURE system, a radically reduced CF expression system containing only the minimum components necessary for transcription and translation [87,88].

An important part of our studies has been devoted to the incorporation of single isotopically labeled residues within HttEx1 homorepeats by combining CF expression with the tRNA suppression strategy. With our approach, we obtain highly simplified spectra that enable the retrieval of residue-specific information of otherwise highly congested spectra. Derivation of backbone chemical shifts is straightforward and can be transformed into structural information reporting on secondary structural information [89,90,91,92,93]. Moreover, access to side chain chemical shifts allows the investigation of the structure and dynamics of systems for which this information could not be extracted using traditional approaches due to the spectral overlap. Importantly, all these structural investigations can be performed independently of the poly-Q length, and therefore, large pathological HttEx1 constructs can be studied at the residue level.

Although the coupling between CF expression and tRNA suppression has been widely used in the past to incorporate non-natural functional groups into proteins [30], the novelty of the approach is the incorporation of natural amino acids [78,79]. This difference imposes some constraints, which have an impact on the protein production. First, it implies a fixed amount of loaded tRNA_CUA_ at the beginning of the expression that cannot be reloaded during or after the reaction. Second, the inherent lability of the ester bond limits the duration of the CF synthesis. The yield of the tRNA suppression experiments is reduced to around 30–40% of non-suppressed CF synthesis, which is in line with previous observations [79,80]. Conversely to the non-suppressed samples, this yield is only moderately affected by the lysate batch. This observation suggests that the quality of the lysate is not the limiting parameter for the yield of tRNA-suppressed samples. In that sense, the use of a heterologous tRNA with a nonsense anticodon and/or the unbalance of the translation components are most probably at the origin of the decrease of the final yield. We attempted to partially correct these problems by adding additional EF-Tu into the CF reactions. We have shown that activated EF-Tu, which binds loaded tRNA and assists its recognition by the ribosome, effectively protects the loaded tRNA_CUA_ in vitro for long periods. However, this protection is not effective in the context of a CF reaction as it slows down the initiation of the synthesis. The ensemble of these experiments shows that the correct concentration balance of all biomolecules is key for the efficiency of translation and that the addition of loaded tRNA_CUA_ in suppression experiments, which probably disrupts this balance, cannot be recovered by additional amounts of EF-Tu alone. However, the simultaneous optimization of multiple CF reaction parameters at once may lead to an improvement of CF suppression yields in a similar way that it improved standard CF reactions [60].

## 5. Conclusions

In this study we have demonstrated that CF expression is a very well-suited platform to produce HttEx1 with tailored isotopic labeling schemes, from uniform to site-specific labeling, by exploiting the composition versatility of the reaction mixtures. This way, the complexity of the spectral features can be disentangled, and specific questions can be addressed by applying the appropriate labeling scheme. The strategies presented, which have been proven to be useful for HttEx1, can be straightforwardly applied to other proteins harboring low-complexity regions. The structure/function relationships of this family of proteins have been largely neglected due to the absence of appropriate strategies for their study. We propose CF expression as the ideal tool to understand the structural bases of crucial biological processes conducted or regulated by low-complexity proteins such as trinucleotide repeat disorders or liquid–liquid phase separation.

## Supplementary Materials

The following are available online at https://www.mdpi.com/2218-273X/10/10/1458/s1. Figure S1: Effect of EF-Tu on Gln-tRNA_CUA_ stability and CF HttEx1 synthesis.
