# Supplementary material for: Robust Cell-Free Expression of Sub-Pathological and Pathological Huntingtin Exon-1 for NMR Studies. General Approaches for the Isotopic Labeling of Low-Complexity Proteins
Source: Biomolecules. 2020 Oct 19;10(10):1458. doi: 10.3390/biom10101458 (PMC7603387; doi:10.3390/biom10101458)
Supplement: Supplementary file 1 [file biomolecules-10-01458-s001.pdf]

SUPPLEMENTARY INFORMATION

Robust cell-free expression of sub-pathological and pathological huntingtin exon-1 for NMR studies. General approaches for the isotopic labeling of low-complexity proteins.

Anna Morató [1], Carlos A. Elena-Real [1], Matija Popovic [1], Aurélie Fournet [1], Karen Zhang [1], Frédéric Allemand [1], Nathalie Sibille [1], Annika Urbanek [1,*] and Pau Bernadó [1,*]

[1] Centre de Biochimie Structurale (CBS), INSERM, CNRS and Université de Montpellier. 29 rue de Navacelles, 34090 Montpellier, France; anna.morato@cbs.cnrs.fr (A.M.); carlos.elena-real@cbs.cnrs.fr (C.E.R); matija.popovic.ri@gmail.com (M.P.); aurelie.fournet@cbs.cnrs.fr (A.F.); kz7@princeton.edu (K.Z.); frederic.allemand@cbs.cnrs.fr (F.A.); nathalie.sibille@cbs.cnrs.fr (N.S.); annika.urbanek@cbs.cnrs.fr (A.U.); pau.bernado@cbs.cnrs.fr (P.B.)

* Correspondence: annika.urbanek@cbs.cnrs.fr; pau.bernado@cbs.cnrs.fr

Figure S1

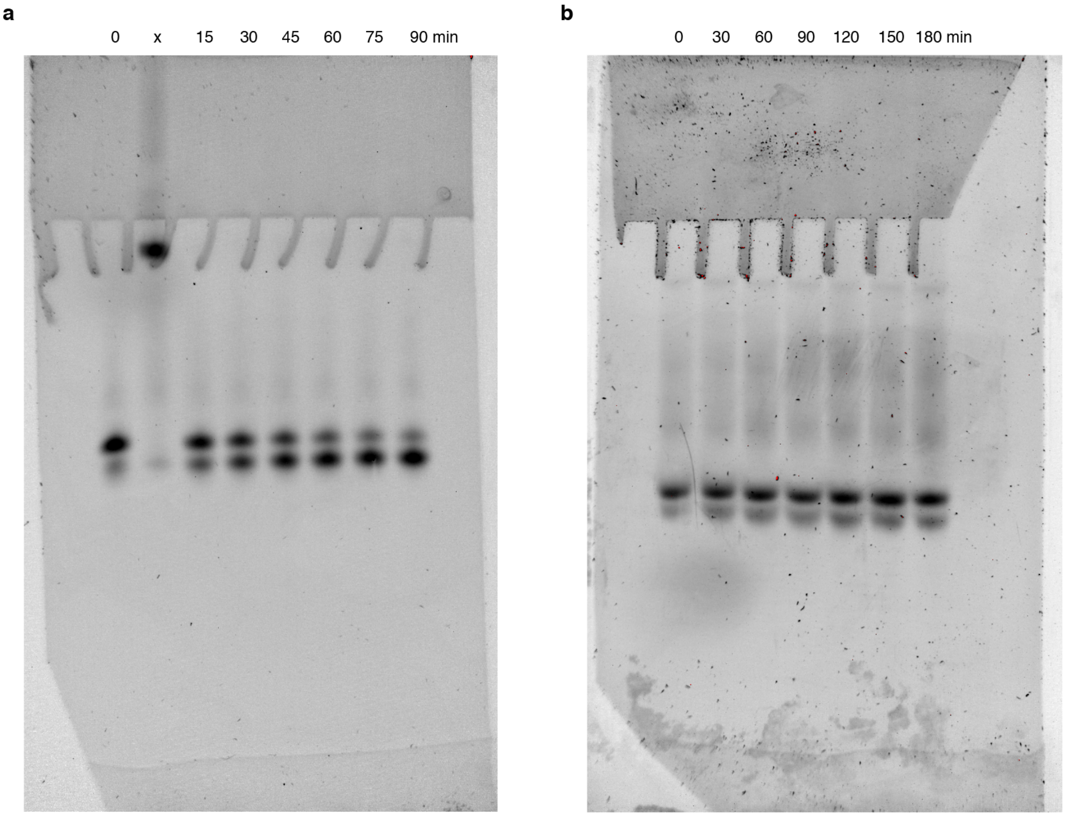

Figure S1. Effect of EF-Tu on Gln-tRNA$_{CUA}$ stability and CF HttEx1 synthesis. Deacylation assay of Gln-tRNA$_{CUA}$ in the absence (**a**) and in the presence (**b**) of a 2x excess active EF-Tu. Original images related to Figure 7 in the main text.
